# Inclusion of *Lacticaseibacillus paracasei* NSMJ15 in broiler diets induces changes in jejunal immune cell population and cecal microbiota

**DOI:** 10.5713/ab.24.0162

**Published:** 2024-06-25

**Authors:** June Hyeok Yoon, Sang Seok Joo, Su Hyun An, Byeong Cheol Ban, Moongyeong Jung, Woonhak Ji, Ji Young Jung, Myunghoo Kim, Changsu Kong

**Affiliations:** 1 Research Institute for Innovative Animal Science, Kyungpook National University, Sangju 37224, Korea; 2 Department of Animal Science, College of Natural Resources and Life Science, Pusan National University, Miryang 50463, Korea; 3 Biological Resources Research Department, Nakdonggang National Institute of Biological Resources (NNIBR), Sangju 37242, Korea; 4 Institute for Future Earth, JYS Institute for Basic Science, Pusan National University, Busan 46241, Korea; 5 Department of Animal Science and Biotechnology, Kyungpook National University, Sangju 37224, Korea

**Keywords:** Broiler, Immune Cell, Microbiota, Probiotics, Short Chain Fatty Acid

## Abstract

**Objective:**

The objective was to investigate growth performance, antioxidant enzyme activity, intestinal morphology, immune cell distribution, short chain fatty acid (SCFA) profile, and microbiota in broiler chickens fed a diet containing *Lacticaseibacillus paracasei* NSMJ15.

**Methods:**

A total of 120 1-day-old Ross 308 male broilers were allocated to 2 dietary treatments in a randomized complete block design. A control group was fed a corn-soybean meal control diet, and an NSMJ15-supplemented group was fed a control diet supplemented with 1 g/kg *L. paracasei* NSMJ15 at the expense of cornstarch. Each dietary treatment had 6 replicates with 10 birds per cage. Growth performance was recorded on day 9. On day 10, one bird representing median body weight was selected to collect serum for antioxidant enzyme activity, jejunal tissue for immune cell isolation and morphometric analysis, and cecal digesta for 16S rRNA gene sequencing and SCFA analysis.

**Results:**

Supplementation of *L. paracasei* NSMJ15 did not affect growth performance, serum antioxidant enzyme activity, and jejunal histomorphology compared to the control group. In the NSMJ15-supplemented group, the population of CD3+CD4+CD8− T cells increased (p = 0.010), while the population of CD3+CD8+TCRγδ+ T cells decreased (p = 0.022) compared to the control group. The *L. paracasei* NSMJ15 supplementation decreased (p = 0.022) acetate concentration in the cecal digesta compared to the control group. The 16S rRNA gene sequencing analysis showed that NSMJ15-supplemented group differentially expressed (p<0.05) 10 more amplicon sequence variants compared to control group without affecting alpha and beta diversity indices of the cecal microbiota. Genera *Mediterraneibacter* and *Negativibacillus* were positively (p<0.05) correlated with CD4+ T cells, while genera *Gemmiger*, *Coprococcus*, *Sellimonas*, *Massilimicrobiota*, and *Blautia* were negatively (p<0.05) correlated with SCFA concentration.

**Conclusion:**

The results of the present study suggest dietary *L. paracasei* NSMJ15 supplementation may increase percentage of CD4+ T cells and decrease acetate concentration in broiler chickens by increasing the differential expression of specific microbial genera.

## INTRODUCTION

Antibiotic growth promoters (AGPs) have commonly been used to prevent disease and improve feed conversion ratio and body weight gain in poultry production. However, excessive use of AGPs has resulted in an increase in antibiotic-resistant pathogens and the presence of antibiotic residues in poultry products [1]. The European Union banned the use of AGPs as feed additives in 2006, the United States in 2014, and China in 2020 [2]. Hence, there is growing interest in the poultry industry to find safe and efficacious AGP alternatives, such as probiotics, prebiotics, and essential oils, to control infectious diseases and limit the spread of resistant bacteria [3].

The beneficial effects of dietary probiotics supplementation in broiler diets have been widely documented in the literature. A meta-analysis study demonstrated that the probiotics supplementation in broiler diets improved body weight gain and feed efficiency of birds [4]. Dietary supplementation of *Saccharomyces boulardii* and *Bacillus subtilis* increased villus height and width, as well as the mRNA expression of tight junction proteins in the jejunum and ileum of broiler chickens [5]. Furthermore, dietary *Lactobacillus paracasei* supplementation alleviated the upregulation of interleukin-8 (IL-8) mRNA expression in the jejunum of broilers challenged with *Clostridium perfringens*. These improvements with probiotics could be achieved by altering the intestinal microbial community and then stimulating host antibody-mediated immunity [6]. However, most of the previous studies have mainly focused on changes in the immune system of birds fed a diet containing probiotics, such as down-regulation of cytokines and immunoglobulins, whereas little attention has been paid to the dynamics of intestinal immune cell populations. Furthermore, limited information is available on gut health, microbiome, and metabolites in the early stage of broilers fed a diet containing *Lacticaseibacillus paracasei*. Therefore, the objective was to investigate the impact of *L. paracasei* NSMJ15 supplementation in a broiler diet on growth performance, antioxidant enzyme activity, jejunal morphology, immune cell distribution, short chain fatty acid (SCFA) profiles, and cecal microbiota in 10-day-old broilers.

## MATERIALS AND METHODS

Experimental procedures were reviewed and approved by the Kyungpook National University Institute for Animal Care and Use Committee, Republic of Korea (approval number: KNU 2021-0213).

### Probiotics preparation

In the previous study, *L. paracasei* NSMJ15 strain, a makgeolli (Korean traditional fermented liquor) isolate, was screened as a potentially probiotic lactic acid bacteria (LAB) with antimicrobial properties against gut pathogens [7]. *L. paracasei* NSMJ15 was cultivated aerobically in de Man, Rosaga, and Sharpe (MRS) broth (BD Difco, Sparks, MD, USA) for 48-hrs at 30°C and then centrifuged at 12,000 rpm for 5 min. The supernatant was discarded, and the pellet was then suspended in 10% (w/v) skim milk (MB cell, Seoul, Korea) as a protectant agent. The mixture was lyophilized using a freeze dryer (FD-8512; Ilshin Lab Co., Ltd, Dongducheon, Korea) to obtain the powder form. The number of colony-forming units (cfu)/g in the lyophilized powder was determined using the plate count method with MRS agar (BD Difco, USA). Skim milk powder was added to the lyophilized powder to a final concentration of 5×10^8^ cfu/kg.

### Animals, experimental design, and dietary treatments

A total of 120 1-day-old Ross 308 male broilers were allocated to 2 dietary treatments in randomized complete block design based on body weight. Each treatment had 6 replicate cages with 10 birds per cage. Corn-soybean meal control diet was formulated without any probiotics and an NSMJ15-supplemented diet was prepared to contain 1 g/kg of *L. paracasei* NSMJ15 at the expense of cornstarch (Table 1). Experimental diets met or exceed the nutritional specifications for Ross 308 broilers [8]. All birds were raised in an environmentally controlled room with continuous lighting. Birds were provided with water and feed *ad libitum* throughout the experiment.

### Growth performance and sample collection

Birds and feed were weighed on days 1 and 9 to measure the growth performance (body weight gain, feed intake, and gain to feed ratio) of birds. Dead birds were removed and weighed daily to correct the growth performance.

On day 10, one bird of the median weight was selected from each pen and euthanized by CO_2_ asphyxiation. Blood samples were collected via the jugular vein into 10 mL tubes. The midpoint jejunal tissues were separated, and 4 cm was used for jejunal immune cell isolation and 1 cm for histomorphometric analysis. The cecal digesta were collected from the ceca for 16S rRNA gene amplicon sequencing and SCFA analyses. Due to a lack of cecal digesta samples to analyze, cecal digesta for SCFA analysis were collected from another bird in a cage and pooled within a cage.

### Serum antioxidant enzyme activity analysis

Serum samples were obtained by centrifugation at 2,000×g for 15 min at room temperature and placed at −20°C before antioxidant activity analysis. Serum samples without dilution were used to determine the concentrations of malondialdehyde (MDA; Cell Biolabs, Inc., San Diego, CA, USA), nitric oxide (NO; BioAssay Systems, Hayward, CA, USA), catalase (CAT; Cell Biolabs, Inc., USA), glutathione peroxidase (GPx; BioAssay Systems, USA) and superoxide dismutase (SOD; BioAssay Systems, USA) as per the manufacturer’s instructions.

### Intestinal histomorphometry

A 1 cm of jejunum tissue was cut and fixed in 10% neutral buffered formalin (Sigma-Aldrich, St. Louis, MO, USA). Tissue samples were embedded in paraffin and cut into 5-μm sections. The sections were stained with hematoxylin and eosin by mounting them on glass slides. Villus height and crypt depth were measured using ImageJ software. And villus height to crypt depth ratio (VH:CD) was calculated.

### Jejunal immune cells analysis

Lamina propria (LP) cells were isolated for further immune cell analysis. Jejunum tissues were gently freed from mucous using a slide glass and washed three times with phosphate-buffered saline (PBS; Thermo Fisher Scientific, Waltham, MA, USA). The washed tissue samples were cut into 0.5 cm pieces and washed with pre-digestion 1 solution, consisting of PBS containing 1 mM DL-dithiothreitol (DTT; Sigma-Aldrich, USA), 30 mM ethylene-diamine-tetra acetic acid (EDTA; Thermo Fisher Scientific, USA), and 10 mM 4-[2-hydroxyethyl]-1-piperazineerhanesulfonic acid (HEPES; Thermo Fisher Scientific, USA) at 37°C for 10 min. Then, tissue samples were washed with pre-digestion 2 solution, which is PBS containing 30 mM EDTA and 10 mM HEPES at 37°C for 10 min. After the washing step, tissues were transferred to 5 mL of 10% fetal bovine serum (FBS) containing RPMI 1640 (GenDEPOT, Barker, TX, USA) and inverted for 2 min. Tissues were digested in 10% FBS containing RPMI 1640 with 0.5 mg/mL collagenase VIII (Sigma-Aldrich, USA) at 37°C for 1 h with shaking at 200 rpm. After the digestion step, isolated LP cells were applied to Percoll (GE Healthcare, Chicago, IL, USA) gradient centrifugation (40% Percoll on the top, 70% Percoll on the bottom).

The isolated LP cells were analyzed by flow cytometry using FACS Canto II (BD, Franklin Lakes, NJ, USA). For flow cytometry analysis, dead LP cells were excluded using Live/Dead fixable dead cell stain (Thermo Fisher Scientific, USA). The analysis was conducted using the following anti-chicken antibodies; anti-CD3 (CT-3; Southern Biotech, Birmingham, AL, USA), anti-CD4 (CT-4; Southern Biotech, USA), anti-CD8 (CT-8; Southern Biotech, USA), anti-TCRγδ (TCR-1; Southern Biotech, USA), anti-MHC II (2G11; Southern Biotech, USA), and anti-Monocyte/Macrophage (KUL01; Southern Biotech, USA). The antibodies were diluted to 1:200 in PBS and incubated for 30 min at room temperature in a light-blocked condition. After the staining process, LP cells were washed using PBS and, they were fixed using 4% paraformaldehyde for 20 min at room temperature, then stored at 4°C before analysis. The analyses were performed using two staining panels: i) MHC II and Monocyte/Macrophages and ii) CD3, CD4, CD8, and TCRγδ T cells.

### Short chain fatty acid analysis in cecal digesta

Approximately 0.5 g of cecal digesta were suspended in 4.5 mL of cold distilled water and mixed with 0.025 mL of saturated HgCl2, 0.5 mL of 25% metaphosphoric acid, and 0.1 mL of 2% pivalic acid. The samples were centrifuged at 1,000×g at 4°C for 20 min. The 1 mL of supernatant was collected and used to measure the SCFA profiles in cecal digesta. The samples were injected into gas chromatography (6890 Series GC System; HP, Palo Alto, CA, USA) equipped with a flame ionization detector and a capillary column (30 m ×0.25 mm×0.25 μm; Agilent, Santa Clara, CA, USA) operated at 50°C in the oven. The inlet and detector temperatures were 180 and 250°C, respectively. Helium was used as a carrier gas.

### The 16S rRNA gene sequencing analysis

To conduct microbiota analysis, total genomic DNA of cecal digesta was extracted using the DNeasy PowerSoil Kit (Qiagen, Hilden, Germany) following the manufacturer’s protocol. Then, the V3–V4 regions of the 16S rRNA gene of chicken cecal digesta were amplified using universal primers and Herculase II fusion DNA polymerase (Agilent Technologies, USA). The polymerase chain reaction (PCR) conditions were as follows: i) 3 min at 95°C; ii) 25 cycles of 30 s at 95°C, 30 s at 55°C, and 30 s at 72°C; iii) 5 min at 72°C. The universal primer set using the Illumina adapter overhang sequence used for PCR was as follows: i) V3-F: 5′-TCGTCGGCAGG TAGAGTAGAGTAGAGCCTACGGGGCWGCAG-3′ and ii) V4-R: 5′-GTCTCGGGGGGGGGTAGAGAGAGAGA GAGAGAGACHVGTATATCC-3′. The PCR products were purified with AMPure beads (Agencourt Bioscience, Beverly, MA, USA). The sequencing library was generated using the Illumina 16S Metagenomic Sequencing Library protocol, and quality was evaluated with quantitative PCR (qPCR) according to the protocol (KAPA Library Quantification kits for Illumina Sequencing platforms). The library was sequenced by using the MiSeq platform (Illumina, San Diego, CA, USA), and (2×300 bp) paired-end reads were generated.

The raw FASTQ data were classified for each sample based on index sequences. Paired-end FASTQ files were produced, and adapter and primer sequences were eliminated from the target gene domains using the Cutadapt (v3.2) software. Sequences with anticipated errors exceeding 2 were rectified using the DADA2 (v1.18.0) tool in R (v4.0.2). To compare microbial compositions, the normalization procedure was carried out with QIIME (v1.9).

Each amplicon sequence variant (ASV) sequence was performed in BLAST+ (v2.9.0) based on a reference database to assign taxonomic information to the most similar microbes. The alpha diversity indices of the cecal microbiota (Chao1, Shannon, and Gini-Simpson) were determined using QIIME with ASVs abundance and taxonomic information. The beta diversity of the cecal microbiota was analyzed by principal coordinates analysis (PCoA) based on the weighted and unweighted UniFrac distance methods using STAMP software (v2.1.3).

### Statistical analysis

Data were tested on the normality over the population by the Shapiro-Wilk test using the UNIVARIATE procedure of SAS (SAS Inst. Inc., Cary, NC, USA). Depending on whether the data satisfied normality, a two-sided two-sample t-test and Wilcoxon Rank-Sum test were performed using TTEST and NPAR1WAY procedures of SAS, respectively. Significance of dissimilarity of microbial communities (beta-diversity) was determined by permutational multivariate analysis of variance test with 999 permutations using Adonis2 in Vegan package of R. Differential expressed ASVs in cecal microbiota was detected using the DESeq2 package of R. Correlation coefficients between the microbial taxonomic abundance and response criteria were determined by Spearman’s rank correlation analysis using the CORR procedure of SAS. The significant level was set at less than 0.05.

## RESULTS

### Growth performance, antioxidant enzyme activity, and jejunal morphology

The effect of *L. paracasei* NSMJ15 supplementation on growth performance of birds is shown in Table 2. Body weight gain, feed intake, and gain to feed ratio were not different between the control and NSMJ15-supplemented groups. Serum antioxidant enzyme activities of birds fed experimental diets are presented in Table 3. Antioxidant enzyme activity of serum in NSMJ15-supplemented group was not different compared to the control group. Supplementation of *L. paracasei* NSMJ15 did not affect the jejunal morphology (villus height, crypt depth, and VH:CD) of birds compared to the control group (Table 4).

### Distribution of immune cells in jejunal lamina propria

**Figure 1:**
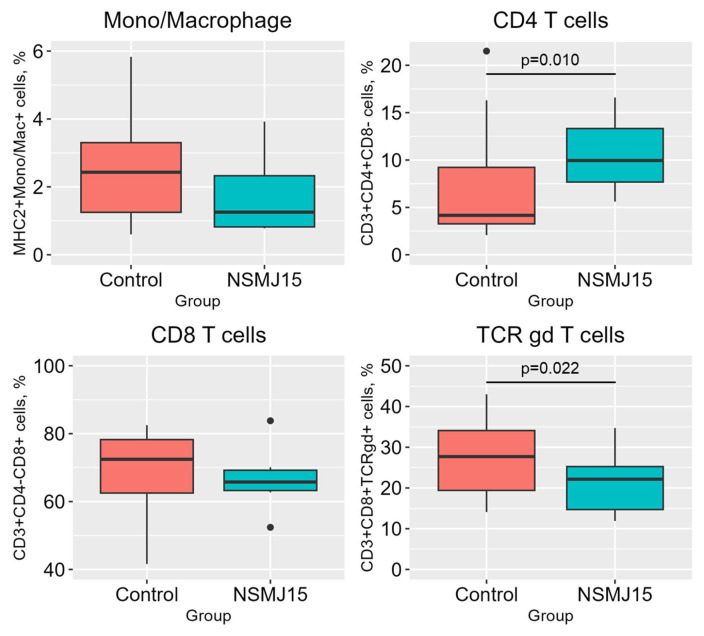
Distributions (%) of monocytes/macrophages and T cells in jejunal lamina propria of 10-day-old birds fed experimental diets. Treatment means were compared using non-parametric test (Wilcoxon Rank-Sum test; n = 6).

The percentages of Mono/Macrophage and T cells in jejunal LP of 10-day-old birds fed experimental diets are presented in Figure 1. The NSMJ15-supplemented group showed increased CD3+CD4+CD8− T cells (p = 0.010) compared to the control group. However, CD3+CD8+TCRγδ+ T cells in the NSMJ15-supplemented group decreased (p = 0.022) more than the control group.

### Concentrations of short chain fatty acid in cecal digesta

The concentrations of SCFA in birds fed experimental diets are shown in Table 5. Results indicated that *L. paracasei* NSMJ15 supplementation decreased (p = 0.022) acetate concentration in cecal digesta compared with the control group. Propionate and isobutyrate concentrations in cecal digesta have a tendency (p = 0.085 and p = 0.052) to decrease as *L. paracasei* NSMJ15 was supplemented in the diet.

### Taxonomic compositions of cecal microbiota

**Figure 2:**
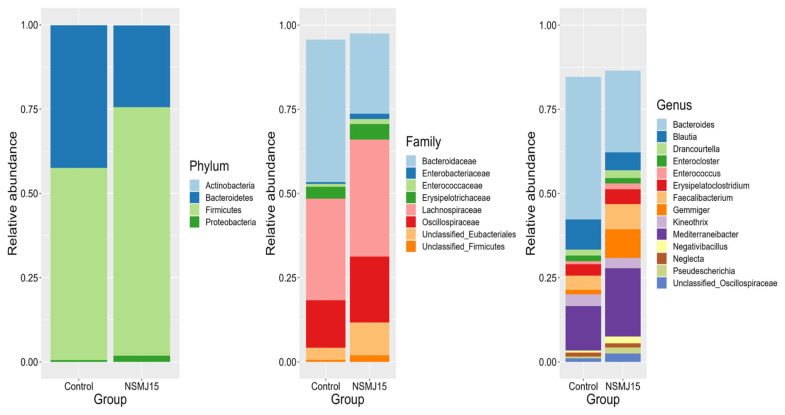
Relative abundance of microbial communities in cecal digesta of 10-day-old broilers fed experimental diets at phylum, family, and genus levels. All phyla, families, and genera that accounted for less than 1% of bacterial taxonomic abundance were classified into the “Others” category. Each color represents a different taxonomic unit (n = 6).

The relative abundance of the microbial community in cecal digesta of 10-day-old broilers from phylum to genus is shown in Figure 2. At the phylum level, *Firmicutes* was the most dominant microbes, accounting for 57.0% in control group and 73.7% in the NSMJ15-supplemented group, followed by *Bacteroidetes* (42.3% for control group and 24.3% for NSMJ15-supplemented group) and *Proteobacteria* (0.5% for control group and 1.8% for NSMJ15-supplemented group). The major cecal microbiota in each level was *Bacteroidaceae*, *Lachnospiracae*, and *Oscillospiraceae* at the family level and *Bacteroides*, *Mediterraneibacter*, and *Blautia* at the genus level.

**Figure 3:**
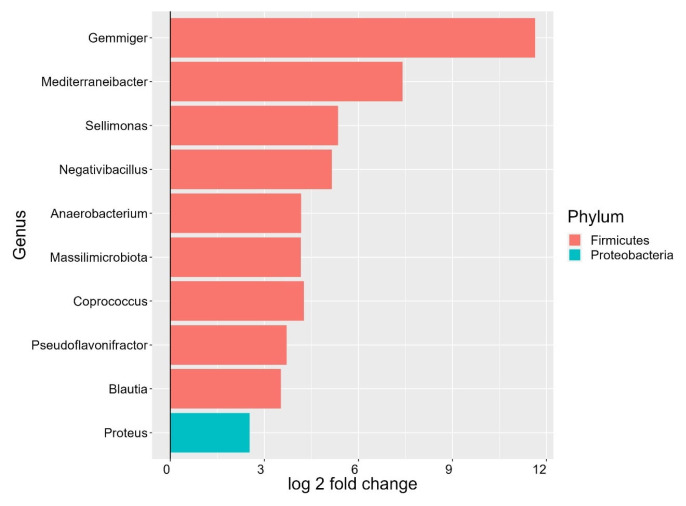
Log_2_ fold change of amplicon sequence variants (ASVs) in the cecal microbiota of 10-day-old broilers fed experimental diets. Values greater than zero indicate that ASVs are enriched in a diet containing *L. paracasei* NSMJ15 compared to the control diet. Different colors represent different microbial phyla (n = 6).

At the phylum level, the relative abundance of *Bacteroidetes* in the NSMJ15-supplemented group was numerically lower than in the control group (Figure 3). However, dietary *L. paracasei* NSMJ15 supplementation numerically increased relative abundance of phylum *Firmicutes* compared to the control group. The *Firmicutes* to *Bacteroidetes* ratio (F:B) also has increased as *L. paracasei* NSMJ15 was supplemented in the diet. At the family level, *Bacteroidaceae* was numerically decreased in the NSMJ15-supplemented group than in the control group. However, relative abundance of families *Lachnospiraceae* and *Oscillospiraceae* numerically increased compared to the control group.

The DESeq2 function was used to distinguish the differentially expressed ASVs in the cecal microbiota in birds fed control and NSMJ15-supplemented diets. At the genus level, ASVs representing genera *Gemmiger*, *Mediterraneibacter*, *Sellimonas*, *Negativibacillus*, *Anaerobacterium*, *Massilimicrobiota*, *Coprococcus*, *Pseudoflavonifractor*, *Blautia*, and *Proteus* increased (p<0.05) in the NSMJ15-supplemented group compared to the control group.

### Alpha and beta diversity of cecal microbiota

**Figure 4:**
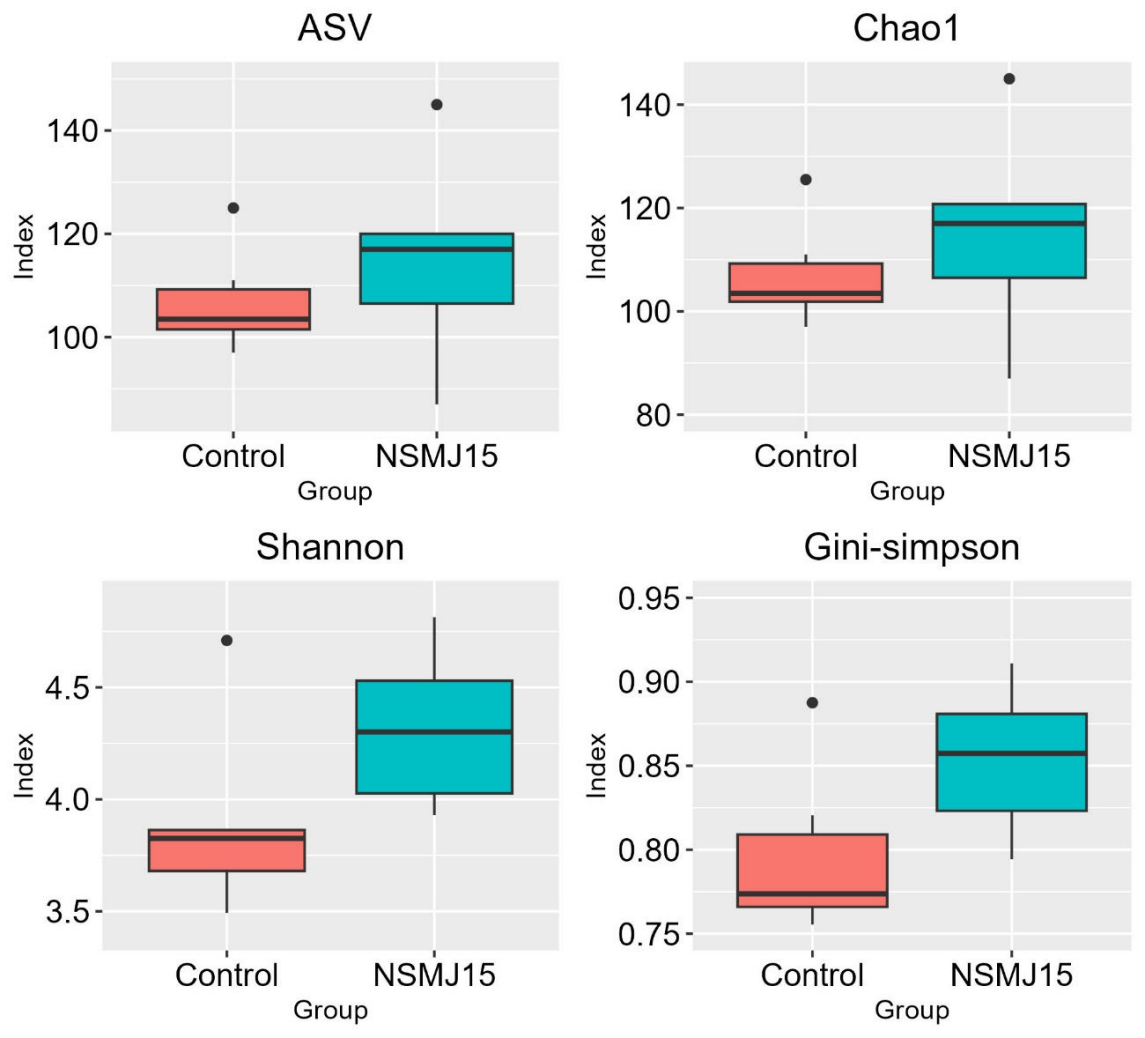
The number of amplicon sequencing variants (ASVs) and alpha diversity indices (Chao1, Shannon, and Gini-Simpson) of cecal microbiota for 10-day-old broilers fed experimental diets. Treatment means were compared using two-sided two-sample t-test (n = 6).

**Figure 5:**
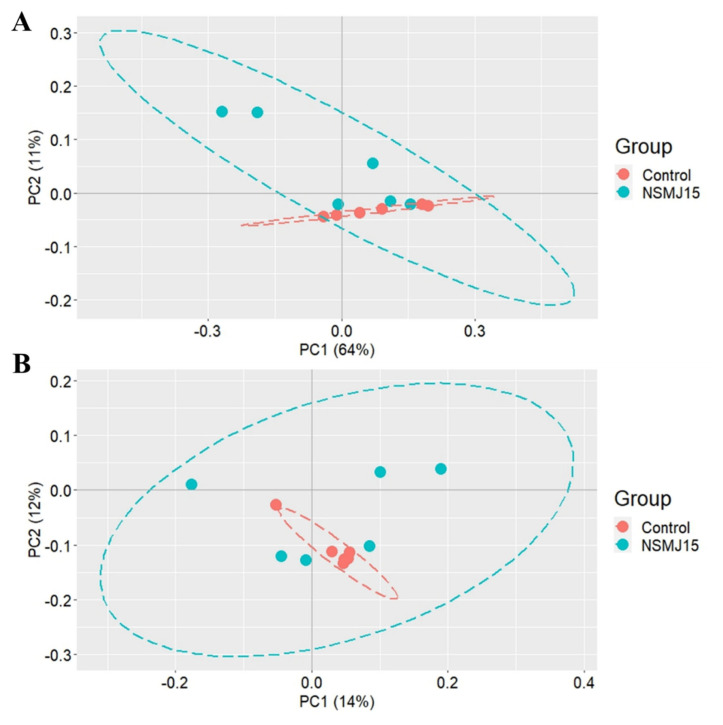
Principal coordinate analysis based on the weighted (A) and unweighted (B) UniFrac distance methods showing dissimilarities (beta-diversity) of cecal microbiota in 10-day-old broilers fed experimental diets. There were no statistical differences in weighted and unweighted UniFrac distance methods between the experimental diets (p = 0.567 and p = 0.853, respectively). Different colors represent different groups (n = 6).

Changes in the number of ASVs and alpha diversity indices of cecal microbiota of 10-day-old broilers fed experimental diets are shown in Figure 4. The number of ASVs and alpha diversity indices (Chao1, Shannon, and Gini-Simpson) of NSMJ15-supplemented group were not different from the control group. Additionally, PCoA plots for beta diversity of cecal microbial community of birds showed that *L. paracasei* NSMJ15 supplementation did not shift the structure and clustering of the microbial communities in the cecal digesta (Figure 5).

### Correlation of cecal microbiota with response criteria

**Figure 6:**
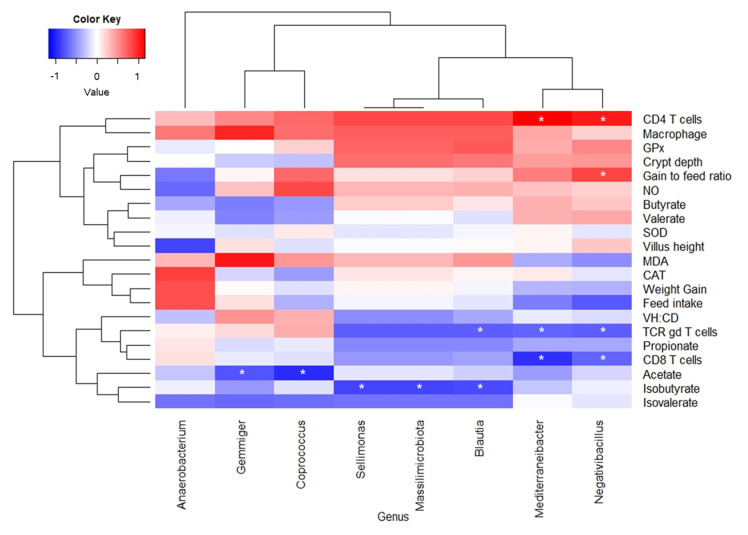
Heatmap of spearman correlation analysis between cecal microbiota and response criteria including growth performance, antioxidant enzyme activity, jejunal morphology, immune cell profile, and short chain fatty acids (n = 6). GPx, glutathione peroxidase; NO, nitric oxide; SOD, superoxide dismutase; MDA, malondialdehyde; CAT, catalase; VH:CD, villus height to crypt depth ratio. * p<0.05.

Spearman correlation analysis was conducted to determine the correlation between the cecal microbial community and response criteria of birds (Figure 6). *Mediterraneibacter* and *Negativibacillus* genera showed a positive correlation (p<0.05) with CD4+ T cells, while there was a negative correlation (p<0.05) with CD8+ T cells and TCRγδ+ T cells. The genera *Gemmiger* and *Coprococcus* showed negative correlation (p<0.05) with the acetate concentration in cecal digesta and the genera *Sellimonas*, *Massilimicrobiota*, and *Blautia* showed a negative correlation (p<0.05) with the isobutyrate concentration in the cecal digesta of birds.

## DISCUSSION

The use of probiotics in the poultry industry has grown over the years as a replacement for AGPs. Various probiotics, such as *Bacillus licheniformis* and *B. subtilis*, could improve growth performance, intestinal histomorphology, resistance against necrotic enteritis, antioxidant capacity, and gut microbiota [9,10]. Some dietary incorporations of *Lactobacillus* species showed inhibition of immune cell inflammation and a balanced fecal microflora [11].

At the onset of an immune response, powerful prooxidants (i.e., hydrogen peroxide) result in oxidative stress, referred to as an imbalance between prooxidants and antioxidants. Excessive prooxidants can damage tissues, including proteins and nucleic acids, and produce large amounts of MDA upon lipid peroxidation. Several antioxidant enzymes, including CAT, GPx, NO, and SOD, could effectively alleviate oxidative stress and maintain the physiological status of the body. Villus height and VH:CD indicate the ability of the intestine to absorb nutrients by increasing the surface area [12]. Dietary *L. paracasei* L1 supplementation was beneficial in improving the body weight gain and feed conversion ratio of broilers [13]. Gyawali et al [14] revealed that the supplementation of encapsulated *L. paracasei* in 42-day-old broilers ameliorated feed conversion ratio, VH:CD, and total antioxidant capacity and decreased MDA concentration. However, in the present study, the supplementation of *L. paracasei* NSMJ15 did not change the growth performance, serum antioxidant enzyme activity, and jejunal morphology compared with the control group. The discrepancy in the present study was likely due to differences in the properties of probiotics (i.e., mode of action, survivability, adhesion capacity, and adaptability) depending on the strain [15]. Additionally, the dose of the supplement, age of the birds, environmental conditions, and dietary composition might have contributed to the discrepant results in the present study.

Previous studies have identified immunoglobulins and proinflammatory cytokines as important indicators reflecting the immune response of animals, which are related to resistance to pathogens and infections [2]. In the present study, we measured the immune cell profiles in jejunal LP of 10-day-old broilers and found a notable increase in the percentage of CD3+CD4+ T cells, whereas there was a decrease in CD3+CD8+TCRγδ+ T cells. CD4 T cells, referred to as helper T cells, coordinate a variety of other cells to activate lymphocytes and innate immune cells against pathogens [16]. Few studies have been conducted to investigate the immune response by determining the distribution of immune cells in response to probiotics supplementation. Wang et al [17] showed that dietary *L. plantarum* P-8 increased CD4+ T cells in small intestine tissues compared to the control group for 42-day-old broilers. Meyer et al [18] reported that the *L. reuteri*-supplemented group had an increased percentage of CD3+CD4+ T cells in the spleen compared to the *L. plantarum*-supplemented group. However, the *L. reuteri*-supplemented group had a lower percentage of CD3+CD8+ T cells in the spleen compared to the *L. plantarum*-supplemented group. The decrease in CD3+CD8+ T cells observed in the previous study might be due to the inverse relationship between CD3+CD4+ and CD3+CD8+ T cells. An increase in the percentage of CD3+CD4+ T cells in the spleen may result in a decrease in the relative proportion of CD3+CD8+ T cells. The results are consistent with the present study, where the increased proportion of CD3+CD4+ T cells in jejunal LP resulted in a numerical decrease in the proportion of CD3+CD8+ T cells, with a particular decrease in CD3+ CD8+TCRγδ+ T cells among CD8 T cells. Based on the aforementioned studies and the findings of the present study, supplementation of *Lactobacillus* probiotics might shift the population of T lymphocytes in favor of helper CD4 T cells in broilers, irrespective of the age of the birds.

The SCFA are the primary products of microbial fermentation in the intestine and serve as a major energy source for colonocytes. The SCFA play a crucial role in intestinal homeostasis, immune responses, and the gut microbiome [19]. Xu et al [2] demonstrated that supplementation with *B. subtilis* or *B. licheniformis* increased acetate and butyrate or butyrate, valerate, and isovalerate concentrations in the cecal contents of broilers compared to the control group, respectively. However, the results of the present study showed that the acetate concentration in the cecal digesta of the NSMJ15-supplemented group was reduced compared to the control group. Therefore, it is necessary to identify changes in the gut microbiome of birds fed *L. paracasei* NSMJ15 to understand the reduced acetate concentration in the present study. Meanwhile, it is possible that the duration of feeding (10 days) may not be long enough to result in an increase in other cecal SCFAs. The profiles of cecal SCFA in the early stage of broilers have not been fully understood, and further studies are needed to deepen the probiotic effects in young chicks.

The gut microbiome is essential for intestinal homeostasis and the functions of host animals, involving the digestion and absorption of feed, immune response against pathogens, and production of metabolites such as SCFA [20]. The ceca of broilers were the most populated parts of the microbial community in the distal intestine, where undigested nutrients could be catabolized by bacterial fermentation. Consistent with the results of the present study, *Firmicutes* was the dominant phylum in the cecal microbiome of broilers, followed by *Bacteroidetes* and *Proteobacteria* [2,13]. In the present study, the relative abundance of *Firmicutes* and F:B increased numerically at the phylum level. *Firmicutes* and *Bacteroidetes* can decompose undigested polysaccharides in the intestinal tract of host animals, enhancing the energy-harvesting capacity [21]. An increased F:B is known to be related to obesity in mice and humans [22]. Huang et al [23] found that the high feed efficiency group had a higher F:B than the low feed efficiency group in broilers. Unlike the current study, Gyawali et al [14] reported that the supplementation of encapsulated *L. paracasei* in broilers decreased the abundance of *Firmicutes* and increased the abundance of *Bacteroidetes* in cecal digesta. The differences might be attributed to the breed, age, and nutritional condition of birds. The taxonomic composition and functions of the microbial community varied depending on the age of birds, and a stable bacterial composition can be established as the age of the bird increases [20].

Based on previous studies, a greater relative abundance of genera *Gemmiger*, *Negativibacillus*, and *Anaerobacterium* was observed with low body weight in broilers [24,25]. However, the genus Proteus, which has been reported to be positively correlated with body weight in birds [26], might compensate for the negative microbial effects associated with low body weight in this study. Additionally, contradictory evidence was also found for SCFA concentrations. The genera *Mediterraneibacter* or *Blautia* are known as butyrate- or acetate-yielding microbes by fermenting glucose and indigestible fiber [27]. However, the genus *Sellimonas* was significantly negatively correlated with butyrate concentration in broiler chickens [28]. The reduced acetate concentration in the present study might partly be due to different populations of microbes producing SCFA profiles in the ceca of birds. Further investigation is warranted to verify the interactions between SCFA concentrations and genera *Mediterraneibacter*, *Blautia*, and *Sellimonas*. On the other hand, the greater relative abundance of genera *Sellimonas* and *Coprococcus* has a beneficial effect on chicken growth by regulating metabolites such as pantothenic acid and menadione, and plays major roles in protecting against *Salmonella Enteritidis* inoculation at the early stage (before 7 days post-inoculation), respectively [29,30]. These results indicate a possibility that an increase in these microbes in the cecal digesta might improve intestinal health against pathogens, therefore intestinal health needs to be further investigated when birds are fed diets supplemented with *L. paracasei* NSMJ15. Regarding the immune response, the genus *Pseudoflavonifractor* showed a negative correlation with the levels of inflammatory factors and nuclear factor kappa-light-chain-enhancer of activated B cells (NF-κB) signaling in lipopolysaccharide-challenged broilers [31]. The relative abundance of the genus *Blautia* was also positively correlated with IL-10 concentration [32]. While previous studies have focused on inflammatory or anti-inflammatory factors to determine immune properties associated with the microbial environment, the present study primarily investigated the relationship between immune cell distribution and the microbiota in the cecal digesta when birds were fed a diet containing *L. paracasei* NSMJ15, as is subsequently discussed.

Although the relative abundance of *Blautia* at the genus level decreased in the NSMJ15-supplemented group (Figure 2), the DESeq2 analysis showed an increase in the genus *Blautia* as birds were fed a diet containing *L. paracasei* NSMJ15. This is because the DESeq2 function identifies differentially expressed ASVs based on the ASV data rather than genus-level data. Therefore, this means that the overall ASVs representing the genus *Blautia* decreased, but the ASV belonging to the specific species in *Blautia* increased. The DESeq2 analysis of the current study revealed that ASV309, *Blautia marasmi*, was increased in the NSMJ15-supplemented group compared to the control group. However, due to limitations in identifying species by 16S rRNA gene amplicon sequencing [33], ASV was classified to the genus level in this study.

Alpha diversity indices, representative of the diversity and richness of the microbial community within the group, were analyzed. The results of beta diversity derived from the PCoA analysis showed the degree of difference between the two groups. Xu et al [13] and Gyawali et al [14] demonstrated that dietary *L. paracasei* supplementation changed the alpha and beta diversity indices compared to the control group. However, in the present study, no microbial community changes were observed within and between experimental treatments of the cecal digesta.

Previous studies have already examined the correlation between the gut microbiome and inflammatory cytokines, metabolites, and growth performance in broiler chickens [24,25,31]. However, the relationship between immune cell distributions and the microbiota in broilers was not well understood, so the present study performed Spearman correlation analysis with immune cells along with growth performance, antioxidant capacity, histomorphology, and SCFA profiles. The results of the current study revealed that differentially expressed genera were mostly correlated with jejunal T cells and cecal SCFA profiles. The reduced acetate concentrations in the cecal digesta of birds fed a diet containing *L. paracasei* NSMJ15 aligns well with the increased abundance of the genera *Gemmiger* and *Coprococcus*, which were negatively correlated with acetate concentration. Positive CD4+ T cells and negative CD8+ and TCRγδ+ T cells correlations with the genera *Mediterraneibacter* and *Negativibacillus* are consistent with the results of immune cell populations in the jejunal LP of broiler chickens. Therefore, the findings of the present study suggest that specific genera of the cecal microbiota modulated by dietary *L. paracasei* NSMJ15 supplementation might influence intestinal immunity and metabolites in broiler chickens.

In conclusion, the findings of the present study indicate that dietary *L. paracasei* NSMJ15 supplementation did not impact the growth performance, serum antioxidant enzyme activity, and jejunal morphology of broilers. However, birds fed on a diet containing *L. paracasei* NSMJ15 exhibited notable differential expression of specific genera of microbes, potentially leading to increased populations of CD4+ T cells and decreased acetate concentrations. Further investigations are needed to elucidate the interactive effects of the cecal microbiota with intestinal immune cells and metabolites in broiler chickens.

## Figures and Tables

**Table 1:** Ingredients and chemical compositions of experimental diets (as-fed basis)

Items	Experimental diets^1)^	

	Control	NSMJ15
Ingredient composition (g/kg)		
Corn	535.44	535.44
Soybean meal	388.00	388.00
Cornstarch	5.00	4.00
*L. paracasei* NSMJ15	-	1.00
Soybean oil	20.00	20.00
L-arginine	0.93	0.93
L-histidine	0.29	0.29
L-isoleucine	1.00	1.00
L-lysine-HCl	3.49	3.49
L-methionine	2.35	2.35
L-cysteine	1.43	1.43
L-threonine	1.43	1.43
L-valine	2.01	2.01
Limestone	10.58	10.58
Dicalcium phosphate	19.05	19.05
Salt	4.00	4.00
Vitamin premix^2)^	2.00	2.00
Mineral premix^3)^	2.00	2.00
Choline chloride	1.00	1.00
Calculated values		
Energy and chemical compositions (g/kg)		
Nitrogen-corrected metabolizable energy (kcal/kg)	2,976	2,972
Crude protein	230.0	230.0
Calcium	9.6	9.6
Non-phytate phosphorus	4.8	4.8
Standardized ileal digestible amino acids (g/kg)		
Arginine	13.12	13.12
Histidine	4.74	4.74
Isoleucine	8.71	8.71
Leucine	15.25	15.25
Lysine	12.80	12.80
Methionine	4.75	4.75
Cysteine	3.96	3.96
Phenylalanine	9.17	9.17
Threonine	8.13	8.13
Tryptophan	2.28	2.28
Valine	10.12	10.12

1) Corn-soybean meal control diet was formulated without probiotics and NSMJ15-supplemented diet was prepared to contain 1 g/kg of *L. paracasei* NSMJ15 at the expense of cornstarch.

2) Vitamin premix supplied per kilogram of diet: vitamin A, 24,000 IU; vitamin D_3_, 8000 IU; vitamin E, 160 mg; vitamin K_3_, 8 mg; vitamin B1, 8 mg; vitamin B_2_, 20 mg; vitamin B_6_, 12 mg; pantothenic acid, 40 mg; folic acid, 4 mg; niacin, 12 mg.

3) Mineral premix supplied per kg of diet: iron, 120 mg; copper, 320 mg; zinc, 200 mg; manganese, 240 mg; cobalt, 2 mg; selenium, 0.6 mg; iodine, 2.5 mg.

**Table 2:** Growth performance of birds fed experimental diets from day 0 to 9^^1)^, ^2)^^

Item	Experimental diets^3)^		SE	p-value

	Control	NSMJ15		
Body weight gain (g/bird)	156	152	4.0	0.411
Feed intake (g/bird)	175	167	5.1	0.162
Gain to feed ratio (g/g)	0.89	0.92	0.018	0.184

SE, standard error.

1) Each of treatment mean represents 6 replicates.

2) Two-sided two-sample t-test was used to compare the treatment means.

3) Corn-soybean meal control diet was formulated without probiotics and NSMJ15-supplemented diet was prepared to contain 1 g/kg of *L. paracasei* NSMJ15 at the expense of cornstarch.

**Table 3:** Serum antioxidant enzyme activities of 10-day-old birds fed experimental diets^^1)^, ^2)^^

Item	Experimental diets^3)^		SE	p-value

	Control	NSMJ15		
Catalase (U/mL)	9.18	9.42	1.182	0.803
Glutathione peroxidase (U/L)	529	484	63.2	0.626
Malondialdehyde (μmol/L)	22.5	21.7	1.80	0.760
Nitric oxide (μmol/L)	7.68	6.48	2.633	0.756
Superoxide dismuatase (U/mL)	1.12	0.92	0.206	0.492

SE, standard error.

1) Each of treatment mean represents 6 replicates.

2) Two-sided two-sample t-test was used to compare the treatment means.

3) Corn-soybean meal control diet was formulated without probiotics and NSMJ15-supplemented diet was prepared to contain 1 g/kg of *L. paracasei* NSMJ15 at the expense of cornstarch.

**Table 4:** Intestinal morphology of jejunal tissues of 10-day-old birds fed experimental diets^^1)^, ^2)^^

Item	Experimental diets^3)^		SE	p-value

	Control	NSMJ15		
Villus height (μm)	469.6	449.6	15.94	0.338
Crypt depth (μm)	107.1	109.9	8.96	0.802
VH:CD	4.53	4.18	0.321	0.462

SE, standard error; VH:CD, villus height to crypt depth ratio.

1) Each of treatment mean represents 6 replicates.

2) Two-sided two-sample t-test was used to compare the treatment means.

3) Corn-soybean meal control diet was formulated without probiotics and NSMJ15-supplemented diet was prepared to contain 1 g/kg of *L. paracasei* NSMJ15 at the expense of cornstarch.

**Table 5:** Cecal short chain fatty acid profiles of 10-day-old birds fed experimental diets^^1)^,^2)^^

Item (mM/g)	Experimental diets^3)^		SE	p-value

	Control	NSMJ15		
Acetate	0.267	0.195	0.0207	0.022
Propionate	0.064	0.035	0.0133	0.085
Isobutyrate	0.037	0.021	0.0054	0.052
Butyrate	0.150	0.163	0.0300	0.646
Isovalerate	0.016	0.014	0.0022	0.171
Valerate	0.014	0.013	0.0017	0.866

SE, standard error.

1) Each of treatment mean represents 6 replicates.

2) Two-sided two-sample t-test was used to compare the treatment means.

3) Corn-soybean meal control diet was formulated without probiotics and NSMJ15-supplemented diet was prepared to contain 1 g/kg of *L. paracasei* NSMJ15 at the expense of cornstarch.
